# Brief intervention to strengthen adherence to ART in prison

**DOI:** 10.1186/1471-2334-14-S4-P13

**Published:** 2014-05-29

**Authors:** Adrian Octavian Abagiu, Rodica Gruia, Laurentia Florentina Ștefan

**Affiliations:** 1 National Institute for Infectious Diseases “Prof. Dr. Matei Balş”, Bucharest, Romania; 2 Jilava Prison Hospital, Jilava, Romania; 3 National Administration of Peniteciaries, Romania

## 

We have learned that the HIV+ prisoners in antiretroviral therapy (ART), frequently discontinue their treatment. That is why we build up and deliver a brief intervention, to see if we can increase their adherence to treatment. We are presenting now the results one month after the intervention, those at 3 month will be available at the congress time.

In the prison hospital Jilava near Bucharest are held the inmates who are HIV+, their number is around 280 and 178 are in treatment. We delivered to 180 of them in series of 30 inmates a 30 minutes session consisting in a short presentation on HIV and the importance of treatment in obtaining undetectable viral loads and then a Q and A session. All of the inmates had access to a written material with almost the same message as the presentation. We than analyze and compare the adherence to treatment in the month before the intervention and the month after the intervention by counting the refused dosages as treatment is delivered strictly supervised by the nurses. We plan to analyze the attrition of the intervention with follow-up at 3 months and 6 months.

As the inmates come and go into the Prison Hospital, we have retained in the analysis only 150 patients that have received the intervention, comparing them with a lot of 50 that didn't, all of them being convicted for at least another year. 120 patients were in ART and 30 were not. In the comparing group 30 were in ART. The demographics of the 2 groups were similar. In the month before the intervention there were 41 refused doses (from 230) and 2 refusals for initiation of therapy. In the comparing group there were 9 refused doses from 55. After the intervention we had less refusals in both groups.

As the adherence was improved after the brief intervention, even though there were positive results in the comparing group that received only the written material, we agreed to deliver the intervention to all the inmates that are HIV positive. The results at 3 months will be available at the National HIV Congress time.

